# Food security and nutrition in the Russian Federation – a health policy analysis

**DOI:** 10.3402/gha.v8.27537

**Published:** 2015-06-24

**Authors:** Karsten Lunze, Elena Yurasova, Bulat Idrisov, Natalia Gnatienko, Luigi Migliorini

**Affiliations:** 1 Department of Medicine, Boston University, Boston, MA, USA; 2 World Health Organization, Russian Federation, Moscow, Russia; 3 Bashkir State Medical University, Ufa, Russia

**Keywords:** nutrition, food security, Russian Federation, non-communicable diseases, health policy

## Abstract

**Background:**

In the Russian Federation (Russia), an elevated burden of premature mortality attributable to non-communicable diseases (NCDs) has been observed since the country's economic transition. NCDs are largely related to preventable risk factors such as unhealthy diets.

**Objective:**

This health policy study's aim was to analyze past and current food production and nutritional trends in Russia and their policy implications for Russia's NCD burden.

**Design:**

We examined food security and nutrition in Russia using an analytical framework of food availability, access to food, and consumption.

**Results:**

Agricultural production declined during the period of economic transition, and nutritional habits changed from high-fat animal products to starches. However, per-capita energy consumption remained stable due to increased private expenditures on food and use of private land. Paradoxically, the prevalence of obesity still increased because of an excess consumption of unsaturated fat, sugar, and salt on one side, and insufficient intake of fruit and vegetables on the other.

**Conclusions:**

Policy and economic reforms in Russia were not accompanied by a food security crisis or macronutrient deprivation of the population. Yet, unhealthy diets in contemporary Russia contribute to the burden of NCDs and related avoidable mortality. Food and nutrition policies in Russia need to specifically address nutritional shortcomings and food-insecure vulnerable populations. Appropriate, evidence-informed food and nutrition policies might help address Russia's burden of NCDs on a population level.

No meu prato que mistura de Natureza!

As minhas irmãs as plantas,

As companheiras das fontes, as santas

A quem ninguém reza…

Alberto Caeiro, “O Guardador de Rebanhos – Poema XVII”, 8 Mar 1914

Availability of and access to a healthy variety of safe food are a prerequisite for healthy nutrition and health promotion. The three leading risk factors contributing to disease burden in the Russian Federation (Russia) are diet, alcohol use, and hypertension (1). A high proportion of disease burden in Russia is attributable to risk factors related to nutrition such as hypertension, hypercholesterolemia, low fruit and vegetable intake, obesity, and harmful alcohol use (1). Nutritional trends are discouraging. For example, the average per-capita consumption of vegetables in Russia fell from 85 kg per year in 1990 (which corresponded to 75% of the US consumption) to 71 kg in 1994 (2). At the same time, the country's burden of premature mortality attributable to non-communicable diseases (NCDs) has increased over the past decade. The decline in fruit and vegetable consumption alone is attributable to a 28% increase in cardiovascular disease mortality in Russia (3).

At the end of the 1980s, leaders of the Soviet Union under Mikhail Gorbachev, then secretary general of the Soviet Communist party, started a movement of reform (*perestroika*) and opening (*glasnost*) of the socialistic planned economy towards free economic markets. These enormous political and economic changes are now known to have ended with the collapse of the Soviet Union in 1991. The collapse had profound effects on the economy and food policy of its successor, the Russian Federation. Currently, the country faces an unprecedented health crisis attributable primarily to NCDs that negatively affects the economic well-being of individuals and households and might become a barrier to economic growth (4). The role of nutrition and food security has not yet been comprehensively analyzed and summarized.

The aim of this analysis is to examine past and present food production and consumption trends in Russia, and to relate these trends to the country's NCD burden. Finally, we suggest potential policy strategies to address malnutrition, including over- and undernutrition, in Russia.

## Methods

**Fig. 1:**
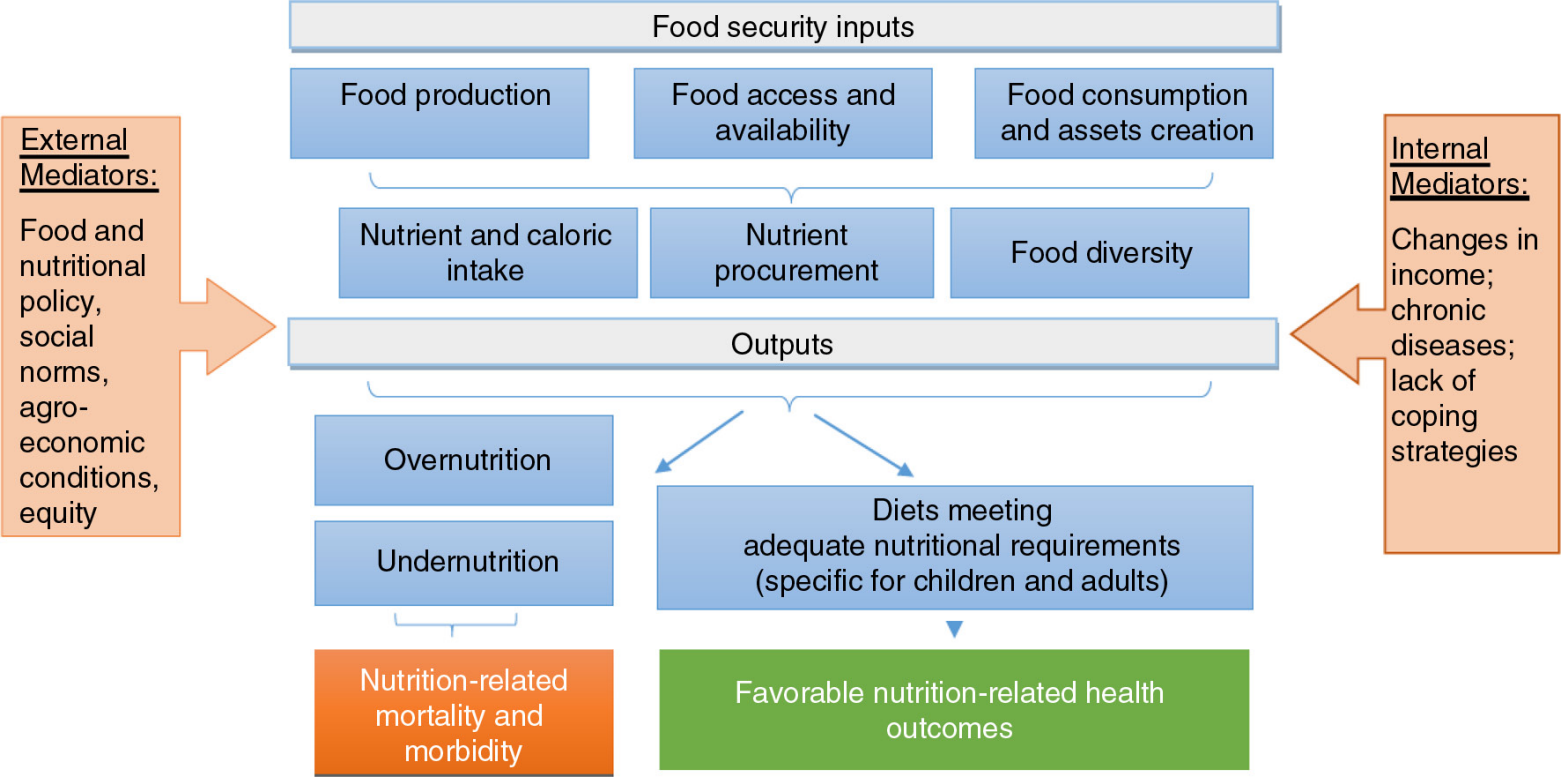
Analytic framework of food security and nutrition in Russia.

This study investigates policy changes in Russia as a single instance (5) from the time of economic transition in the 1990s to the present, based on a narrative review to allow for a comprehensive coverage of the topic. Narrative reviews summarize diverse subject areas and a variety of research designs on a given topic, based on the background of the reviewers’ experiences framed by existing theories and models (6). Our analytic approach was informed by an analytic framework on food security and nutrition as summarized in Fig. 1.

We used Medline/PubMed as our primary electronic reference database and conducted secondary searches in EMBASE and Web of Science for English and Russian language sources reported between January 1990 and July 2014 using a combination of following keywords: Russia, Russian Federation, Soviet Union, nutrition, food, food security, agriculture, production, macronutrient, micronutrient, and policy. We also conducted a similar search using standard search engines (Google and Google Scholar) and searched the bibliographies of the retrieved articles written by experts in food and nutrition in the Russian country context.

We selected studies according to the following criteria: 1) reporting of food availability, production, and/or consumption, 2) reporting of nutrition indicators (body mass index, BMI, weight, etc.), and 3) reporting of dietary or food habits. Drawing from identified peer-reviewed and grey literature, our framework uses primary concepts of food security analysis, that is, availability of food, access to food, and nutrition (7).

## Results

### Food availability and food access policies were highly regulated during the times of the Soviet Union

Russia was the largest country of the Soviet Union and had vast potential resources to supply its population with ample quantities of fresh meats and dairy products. These were consumed at affordable prices due to the central planning system which coordinated and assured distribution to the population (7). Since the 1960s, the Soviet Union expanded its livestock sector, an approach adopted in virtually all of its Eastern European satellite countries. Communist leaders pushed for consumption patterns to closely resemble the ‘Western diet of progress’, notably including meat consumption (8). By the end of the Soviet Union, livestock and meat production had increased by 50% (2). The government aimed to improve living standards by increasing consumption of high-value livestock products without consumers having to pay the high cost of livestock production. Socialistic food markets were highly regulated, and the Soviet Union used 11% of its GDP to subsidize consumer prices, so that livestock cost the consumer only about half of the actual production costs (9).

Early Soviet policies pushed for the consumption of livestock products. The recommended daily intake for protein set by the former Soviet Union's Ministry of Health was almost twice that of Europe and North America, creating the erroneous belief that such high intakes of fat and protein are necessary for maintenance of health (10). The total recommended daily amount of calories for a Soviet person ranged from 2,800 to 3,600 for men and from 2,400 to 3,100 for women, depending on their occupation (11). People of all ages in Russia suffered from an excess intake of protein and fat rather than a deficiency, a trend that continues to the present day despite relatively high prices for these commodities and partially explained by reference to the old standards established by the former Soviet Union's Ministry of Health (10).

Thus, the period between 1960 and 1989 saw an overall shift in food commodity consumption from a diet of starchy staples such as bread and potatoes to one with high amounts of meat, dairy products, and sugar (12). However, these consumption data incompletely reflect Russian population's diet. The Soviet Union's large agricultural production was distributed unequally, leading to the ubiquitous lines of customers in front of almost-empty food stores. In order to ensure food security, the population relied heavily on efficient social trade networks and home production on small plots on their *dachas* (weekend homes) out of economic necessity (12).

### Food availability and access declined during the economic transition in Russia, but caloric intake remained constant

*Glasnost* and *perestroika*, the globalization of Russia's political and economic system, brought about a drastic decrease in the country's gross national income, along with an increase in population poverty (13). Later analyses suggest that the rapid mass privatization during the economic transition was also associated with an increase in short-term adult male mortality rates (14). While adult death rates have been constantly decreasing in Western Europe since the 1960s, in the Russian Federation, they remained stable between the mid-1960s and mid-1980s; in the 1990s, death rates increased in Russia, leading currently to a life expectancy at birth in Russia that is more than 12 years lower than that in Western Europe (15).

The collapse of the Soviet Union and the ensuing introduction of free markets had dramatic economic implications for the Russian population, and population poverty rose (13). From 1992 to 1998, the proportion of the Russian population living below poverty level increased from 12 to 46%, while gross national income decreased by 22% (12). The most immediate impact on food availability and nutrition was the decline in the production and inventories of livestock; livestock products have high-income elasticity of demand compared to other foodstuff, making its demand highly responsive to income changes (9). This consequently resulted in a reduced demand and output of feed grains (16). The Kremlin adopted its first policy ‘Concept of Federal Healthy Nutrition Policy in Russia by the Year 2005’ in 1998. It emphasized aspects of a healthy diet, regular physical activity, prevention of obesity, and food safety.

While Russia's overall agricultural production fell by 29% (and the import of grain and meats increased), the overall food consumption in Russia stayed constant. According to data from the Russian State Commission for Statistics (Goskomstat) household surveys, the average energy availability remained at about 2,900 kcal per-capita per day from 1990 to 2000, well above the Food and Agricultural Organizations of the United Nations (FAO) energy requirement guidelines of about 1,900 kcal per-capita per day (16). Other important sources of dietary indicators are the University of North Carolina's Russian Longitudinal Monitoring Survey (RLMS), as well as data from FAO, UNICEF, and WHO, which likewise found that actual energy and macronutrient intake remained stable during the economic transition (12).

Several factors have contributed to Russian consumers’ stable food access and maintenance of caloric intake. While agricultural production was falling, incomes were decreasing, and food prices were rising (Table 1). Both rural and urban families made use of private land plots (*dachas*) to produce food for their own consumption. Households also increased the proportion of their income spent on food, and individuals changed their dietary habits away from expensive (and less healthy) livestock towards vegetables and starchy foods.

**Table 1:** Consumer behavior to maintain caloric intake

Indicator	Early transition year 1992	Late transition year 2000
Private plot production (% of total)	31	57
Household expenditures on food (% of total)	38	50
Fat from livestock product (% of total fat available)	76	57

Adapted from Ref. (16).

Much of the literature on food security in Russia focuses on the debate of changes in agricultural policy. Changes in agricultural production and consumption, adjustment of consumer purchase behavior following the abolition of massive consumer subsidies, and their replacement by actual scarcity prices for agricultural products were all likely inevitable consequences of the economic reforms. FAO asserts that these trends also reflect the comparative advantage of Russia in the production of crop rather than livestock (16).

Although on average food consumption remained above FAO energy recommendations (16), during short periods in the early years of the economic transition, Russians experienced hunger. A 1993 survey conducted by the humanitarian aid organization CARE classified 70% of households, 77% of women, and 32% of children as hungry (defined as a positive answer to items using the Radimer/Cornell hunger scale) (17). For the duration of 5 years, the USA and EU delivered food aid to Russia. In 1999, due to unfavorable weather conditions the harvest was the lowest in 50 years, and food donations to Russia from 1999 to 2000 surmounted all US and EU food aid to Africa combined (9).

During the economic crisis from 1992 to 1998, the proportion of overweight among children aged 6–18 years decreased (from 15.6 to 9.0%), while underweight increased (from 6.9 to 8.1) (18). In fact, over- and underconsumption coexisted: the majority (58%) of Russian families with an underweight individual also had at least one overweight family member (19). However, the recent economic recovery primarily fueled the overweight and obesity epidemic in Russia.

### Current food insecurity in Russia is low, but food availability affects some vulnerable groups

Due to the high proportion of private food production and the high caloric intake at baseline, Russia's currently food inadequate population by FAO standards is estimated to be low, at 5–6%, a number that compares favorably to other transitional (7%), Asian (16%), or African countries (33%) (16).

#### Changes in income and purchasing power

The increase in income inequality resulting from the economic transition added to the vulnerability of affected households. The country's later macroeconomic growth over the past 10 years contributed to a reduction of poverty and narrowed this gap by reducing poverty rates from 2.1% in 2005 to 1.0% in 2013 (20), but certain socioeconomic groups remain food insecure. Those who do not have the coping mechanisms available shown in Table 1, such as those without private land plots (*dachas*), as well as the unemployed, lost their purchasing power to maintain a healthy diet.

Food access and dietary choices also depend on socioeconomic circumstances. In Russia, socioeconomic hardship primarily affects households with children, people with disabilities, or unemployed individuals (21) and older people on pensions with low incomes. Similar to energy intake and diet, education and income have been shown to have a strong effect on obesity and BMI (22).

#### Domestic food production and legislation

After its accession to the World Trade Organization, Russian food legislation has increasingly been brought into compliance with international norms and standards. Russia continues to coordinate policy reforms with the Eurasian Economic Community and with the European Union, its primary trade partner. The state bodies responsible for control and supervision of food quality and safety in Russia include *Rostekhregulirovaniye* (Federal Agency for Technical Regulations and Metrology) in the Ministry of Industry and Power, Rosselkhoznadzor (Federal Agency for Veterinary and Phytosanitation) in the Ministry of Agriculture, and Rospotrebnadzor (Federal Agency for Consumers Protection and Human Welfare) in the Ministry of Public Health and Social Development (23). *Rospotrebnadzor* oversees the domestic foodstuffs market in Russia based on federal law ‘Hygienic Requirements for Food Safety and Nutritional Value’ (SanPiN 2.3.2.1078-01 and later amendments) and more than 700 state laws and standards and governmental orders regulating food production in Russia. Russian standards partly even exceed EU regulations (e.g. for safe tetracycline concentrations or for food additives in baby food) or food safety standards set by FAO/WHO in Codex Alimentarius (23).

In 2010, the Russian President signed into effect ‘Russia’s Food Security Doctrine’ (24), a framework that mandates a minimum domestic production requirement for Russia's agricultural output in grain and potatoes (90%), milk and dairy products (90%), meat and meat products (85%), and sugar, vegetable oil, and fish products (80%). The policy aims at the independence of domestic production and a guarantee for food safety. The FAO found that Russia's dependence on imported food declined during the economic transition for all commodities except for meat, and confirmed that food availability and food access are already up to international standards even in outlying areas (7).

#### Food diversity, vegetable and fruit production

Russia's food environment falls short in offering healthy choices at affordable prices. The availability of vegetables and fruits is lowest in Russia compared to other countries in the WHO European Region (25). To account for about 30% of vegetables and fruit production which are usually lost to spoilage, waste, or destruction, national average per-capita production or import should be accordingly higher than the WHO recommendation of 400 g intake of fruit and vegetables per person per day (26). Yet, average domestic production of fruit and vegetables in Russia is considerably below the recommended dose. This is largely attributable to the unfavorable climate for fruit production in most of the Russian Federation and the insufficient availability through imports. The country's food supply is limited by the fact that about 70% of Russia lies in the permafrost zone and more than 70% of its territory in a risk zone for agriculture. Consequently, while in the autumn most Russians (75% males and 81% females) consume the recommended level of 400 g, less than half do so during the other seasons (18).

Like in other European countries, Russians with higher education and occupational class have a higher intake of fruit than those of lower socioeconomic status (19). In fact, in a large-scale multinational European cohort study, the Russian sample showed the strongest positive association between fruit consumption and socio-economic factors (27).

#### Nutrient procurement and dietary choices

**Fig. 2:**
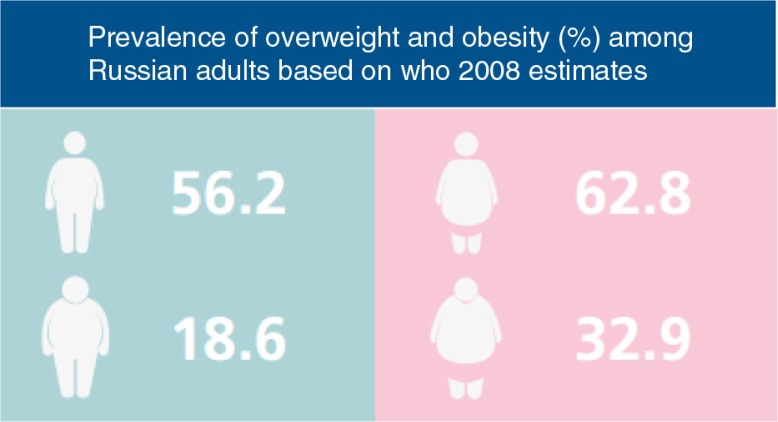
Women in Russia have higher rates of overweight and obesity than men. Source: From Ref. (29).

**Fig. 3:**
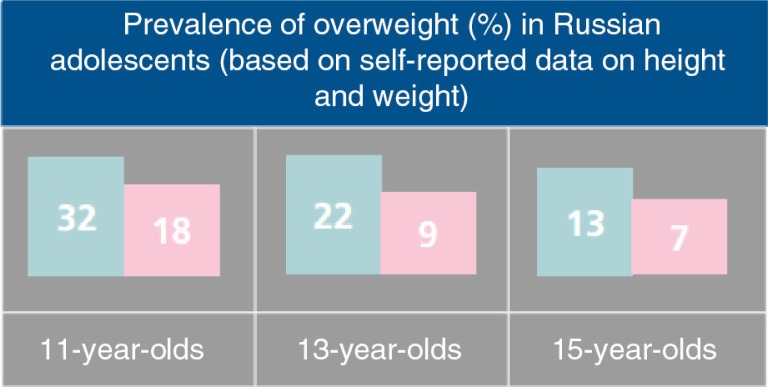
Boys in Russia have higher rates of overweight and obesity than girls. Source: From Ref. (29).

Rather than food insecurity with inadequate energy intake, most Russians face nutritional problems linked to unhealthy diets. Similar to the situation during the Soviet era, preeminent nutritional problems are overweight and obesity. According to latest estimates from WHO, almost 60% of Russia's adult population are overweight, and more than one in four (26.5%) is obese. The prevalence of overweight and obesity in Russia has been increasing over the past three decades (28). In adults, it is higher among women (Fig. 2), whereas in childhood, rates are higher among boys (Fig. 3).

Compared to their Central and Eastern European counterparts, Russians have the highest mean energy intake, with total fat as the main source, mostly provided by monounsaturated fat (18). Assessing the Healthy Diet Indicator, a measure developed using WHO guidelines for the prevention of chronic diseases ranging from a score of 0 (worst) to 7 (best), Russia scores a mean of 1.5 (standard deviation, 0.8), which compares unfavorably to the already low European mean scores of 2.5–3.4 (18).

These unhealthy diets create risk factors for various cardiovascular diseases, cancers, chronic diseases such as diabetes and osteoporosis, and many more. Attributable to the Russian diet that has remained high in unsaturated fat and salt, and low in fruit and vegetables particularly outside harvest season, disorders of the circulatory system increased by 18%, endocrine disorders by 18%, and neoplasm by 16% during the economic transition (30).

Although trends indicate that the overall consumption of high-fat livestock product and sugar has decreased (9), people in Russia still consume too much saturated fatty acids, too much sugar, too much protein, and not enough complex carbohydrates or pulses and nuts (18). High-fat, high cholesterol, low fiber diet due to the low consumption of fruits and vegetables, high consumption of dairy, meat, sugar, and alcohol may have contributed to the high levels of overweight and obesity in Russian adults. With an average BMI in Russia of 26.5 for men and 29.8 for women, both genders exceed the normal range of 18.5–25 and, on average, fall into the overweight category (21). Overweight and unhealthy nutrition are underlying risk factors for cardiovascular diseases and cancers, which together with injuries account for 78% of deaths in Russia (4).

#### Children and nutrition

The current preeminent nutritional problem for children of all ages in Russia is overweight. Latest available data from the Health Behaviour in School-aged Children survey suggest that overweight in Russia is higher during early adolescence, as up to 32% of boys and 18% of girls among 11-year-olds were overweight (31), rates which decrease with age during adolescence (Fig. 3). Compared to the high prevalence of overall adolescent obesity and overweight in the US (combined prevalence of 25.4%) and low rates in China (combined prevalence, 7.0%), Russia had a moderate combined prevalence of 16% according to the RLMS (32). However, these trends seem to increase. A recent study on the global burden of overweight and obesity estimated that overweight and obesity rates have increased to 22% in boys and 19% in girls (28).

The few available data on breastfeeding indicate that rates have remained low (33). According to subnationally representative data from 2000, the prevalence in Russia of any breastfeeding at 6 months of age was 47% (31). Overnutrition in Russian children may be related to late initiation and low rates of breast feeding (30), to deficiencies in weaning practices and to improper complementary feeding (16).

Stunting, that is low height for age, is usually the result of chronic malnutrition early in life, a concept that has recently focused on the first 1,000 days of life from conception to 2 years of age. Stunting in Russian children under 5 years of age has been estimated to be as high as 13% (7). Micronutrients are essential to ensure appropriate length growth. Since infants and older children in Russia tend to be overweight, the country's high stunting rate is better explained by a deficiency in micronutrients. Iodine, fluoride, and iron deficiency with subsequent anemia are common in Russian children, as are vitamin deficiencies, particularly in vitamin C (16).

#### Vulnerable populations

Russia is currently experiencing an exponentially increasing double epidemic of injection drug use and HIV infection. This has created a rapidly growing but mostly overlooked vulnerable population at risk for malnutrition, people who inject drugs (PWID) and people living with HIV (PLWH). Malnutrition and food insecurity are exacerbated in these patient groups but are currently not routinely addressed in the care of PWID or PLWH in Russia.

Among HIV-infected PWID, food insecurity increases the risks of HIV transmission to their non-infected sex partners. The increased risk is mediated by nutritional deficiencies, which further compromise immune status and increase transmission risk per unsafe sex act. In addition, poverty-related food insecurity triggers transactional sex and needle-sharing practices. Food-insecure, HIV-infected PWID also have less access to health services including ART. Even among those on ART, pharmacokinetic efficiency of the drugs remains low due to reduced energy intake.

Russia's healthcare system is highly fragmented and does not address nutritional problems in the vulnerable population of HIV-infected PWID. While there are reliable estimates on the combined epidemic of drugs and HIV in the Russian Federation, there are currently no published data on nutritional status and food insecurity in these key populations. Studies from other concentrated HIV epidemics indicate high levels of food insecurity and poor nutritional status among PWID regardless of HIV status (34). In the context of the country’s existing micronutrient malnutrition, Russian PWID might be particularly vulnerable. They face a constant challenge of an irregular life style dominated by drug seeking behavior, low incomes and high unemployment rates (35).

In Russia, injection drug use (which consists almost exclusively of injectable opiates) has been driving the HIV epidemic (36) and has been increasing since the break-up of the Soviet Union and the following political and socioeconomic turbulences (37). The United Nations Office on Drugs and Crime estimates that more than 2 million Russians, or 2.3% of the adult population, use opioids (38). In Russia, 14.4% of PWID are HIV infected (39), a proportion that has reached 60–82% in some urban centers (40). This is attributable to rising HIV incidence rates, which among PWID in St Petersburg have almost tripled from 4.5 to 14/100 person years over the past 5 years alone (41). HIV prevalence has been growing at alarming rates in Russia and become a serious public health problem particularly for drug users and their sex partners (42). HIV prevalence in Russia has increased exponentially from less than a thousand cases a little over a decade ago to close to 1 million people (1.1% of adults) according to current authoritative estimates from international organizations (43, 44), and 665,000 HIV patients are registered with the government (45).

Since the advent of the HIV epidemic, research in various settings has shown that HIV patients suffer from complex nutritional problems, notably wasting and micronutrient deficiencies (46). Many PWID are affected by weight loss, wasting, and micronutrient insufficiencies. While the Russian health system offers health services that could address these problems, health institutions are difficult to navigate for PWID, whose nutritional issues are mainly those related to HIV infection. Most vulnerable are individuals in whom both the HIV and drug epidemic overlap and who subsequently suffer from synergistic nutritional problems (47).

Malnutrition in HIV-infected PWID has various causes, including malabsorption related to both HIV infection and ART, as well as decreased dietary intake due to gastrointestinal pathologies, irregular lifestyle and limited access to appropriate food (both often related to drug use), or mental illness. An HIV-induced hypermetabolic state may also contribute to malnutrition in this population (48).

Addressing nutritional issues in a specifically vulnerable population, that is PWID in the context of a concentrated HIV epidemic, with particular attention to micronutrient needs, has been a neglected aspect of a politically charged public health problem in Russia. Food and nutritional support for AIDS prevention among HIV-infected PWID should include not only brief screening and comprehensive assessments of nutritional status, counseling and education, and various food support interventions, but also advocacy and appropriate nutrition and HIV policies (49).

## Discussion

Russia's burden of overweight contributes to the existing health crisis in the country. While gross national income has recovered over the past years, life expectancy and population size continue to decline. Appropriate national policies need to address the problem of malnutrition, which in Russia includes not only overnutrition but also micronutrient deficiencies and to a lesser extent undernutrition. Policies should also be tailored to the most vulnerable, that is women and children, populations in lower socioeconomic strata, and focus on high-risk groups such as HIV patients and PWID.

To face the nutritional burden, Russia's national food and nutrition policy needs to go beyond agricultural policies to have a potential public health effect. Changing food prices alone is unlikely to substantially reduce BMI and obesity in Russia, as elasticities of BMI with respect to food prices are low, mostly smaller than 0.01 (50). The burden of malnutrition (including over- and undernutrition) carries the risk of exacerbating the problem of undernutrition when solely addressing excess energy intake. Policies should therefore target healthy nutrition and address not only insufficient availability of fresh fruit and vegetables but also their intake through cultural changes towards healthier habits.

Russian public health experts attribute the causes of malnutrition in Russia to the limited public awareness of healthy eating habits and to insufficient training of providers in delivering adequate nutritional counseling. They have argued that healthier dietary habits have to start early in life and need to be rooted in the communities, for example through sufficiently funded nutrition groups. Baby-friendly hospitals are a potential implementation site for programs in Russia to improve mother and child nutrition, focusing on improving breastfeeding practices and healthy maternal nutrition. Although established since 1996 in Russia, only a small number of deliveries (16%) occur in baby-friendly hospitals (51). Mother and child health interventions have been shown to substantially improve breastfeeding rates in Russian maternity hospitals (52).

Policies need to specifically address Russia's food-insecure minorities. While there has been some international attention for people of lower socioeconomic status and populations living in the economically disadvantaged peripheral regions of the vast Russian Federation, there has been no focus so far on nutritional high-risk groups and key populations such as PLWH and PWID. One of the reasons is the immense stigma associated with these conditions. To overcome policy resistance against evidence-based management of these potential target groups, political, social, and religious leaders will have to overcome their own prejudices and actively address the stigma of HIV and drugs. It is a mandatory prerequisite for national food policies to bring progress for Russia's nutritionally vulnerable and food-insecure minorities.

Policy resistance against limiting a flourishing food industry and lack of funding for public health nutrition programs are expected barriers towards halting and reversing current nutritional trends. Similar to experiences of Western countries, Russia's school-aged children face an environment rich in advertisements and offerings of calorically dense, processed food and soft drinks, like candy, snacks, and sugared beverages (53). However, appropriate regulations such as taxes specifically on sugared beverages and curricula inclusive of healthy nutrition habits could help school-aged children's cope with their exposure to these so-called increasingly obesogenic environments. In addition, school feeding programs, positive remnants of Soviet times, can promote both healthy diets and physical activity.

The Russian Government's key policy ‘Concept 2020’ and its framework for the national health strategy ‘Healthcare Development Concept to 2020’ (54) aims to increase qualification of medical care providers and to create a comprehensive system which increases their performance in overall provision of health care. Healthy nutrition has to be a central component of health systems modernization efforts. It is a central element of prevention, as reflected in a Russian proverb: Eсли питаться правильно, то врачи будут не нужны, а если неправильно, то врачи уже не помогут. [If you eat right, you won't need doctors; but if you don't eat right, the doctors won't be able to help.]

## Conclusion

Despite economic instabilities that accompanied Russia's economic transition, the country fares better in food adequacy than many other countries in the area. Russia's main food-related challenges are unhealthy nutritional habits, closely related to unhealthy alcohol use.

Changing lifestyles and nutritional habits is certainly a complex process and demands a comprehensive approach championed by political and societal leadership, translated to the population through evidence-based programs. A public health approach, that is a population-based shift towards healthier nutrition could not only relieve Russia's premature mortality burden, but also its immense economic effects.
